# Comparative assessment of net CO_2_ exchange across an urbanization gradient in Korea based on eddy covariance measurements

**DOI:** 10.1186/s13021-019-0128-6

**Published:** 2019-09-11

**Authors:** Je-Woo Hong, Jinkyu Hong, Junghwa Chun, Yong Hee Lee, Lim-Seok Chang, Jae-Bum Lee, Keewook Yi, Young-San Park, Young-Hwa Byun, Sangwon Joo

**Affiliations:** 1 Ecosystem-Atmosphere Process Laboratory, Department of Atmospheric Sciences, Yonsei University, Yonsei-ro 50, Seodaemun-gu, Seoul, 03722 South Korea; 2 National Institute of Forest Science, Seoul, South Korea; 3 National Institute of Environmental Research, Incheon, South Korea; 4 Korea Basic Science Institute, Cheongju, South Korea; 5 National Institute of Meteorological Sciences, Jeju, South Korea

**Keywords:** Urbanization, Net CO_2_ flux, Monsoon, Urban, Suburban, Forest, Cropland, Urban vegetation, East Asia

## Abstract

**Background:**

It is important to quantify changes in CO_2_ sources and sinks with land use and land cover change. In the last several decades, carbon sources and sinks in East Asia have been altered by intensive land cover changes due to rapid economic growth and related urbanization. To understand impact of urbanization on carbon cycle in the monsoon Asia, we analyze net CO_2_ exchanges for various land cover types across an urbanization gradient in Korea covering high-rise high-density residential, suburban, cropland, and subtropical forest areas.

**Results:**

Our analysis demonstrates that the urban residential and suburban areas are constant CO_2_ sources throughout the year (2.75 and 1.02 kg C m^−2^ year^−1^ at the urban and suburban sites), and the net CO_2_ emission indicate impacts of urban vegetation that responds to the seasonal progression of the monsoon. However, the total random uncertainties of measurement are much larger in the urban and suburban areas than at the nonurban sites, which can make it challenging to obtain accurate urban flux measurements. The cropland and forest sites are strong carbon sinks because of a double-cropping system and favorable climate conditions during the study period, respectively (− 0.73 and − 0.60 kg C m^−2^ year^−1^ at the cropland and forest sites, respectively). The urban area of high population density (15,000 persons km^−2^) shows a relatively weak CO_2_ emission rate per capita (0.7 t CO_2_ year^−1^ person^−1^), especially in winter because of a district heating system and smaller traffic volume. The suburban area shows larger net CO_2_ emissions per capita (4.9 t CO_2_ year^−1^ person^−1^) because of a high traffic volume, despite a smaller building fraction and population density (770 persons km^−2^).

**Conclusions:**

We show that in situ flux observation is challenging because of its larger random uncertainty and this larger uncertainty should be carefully considered in urban studies. Our findings indicate the important role of urban vegetation in the carbon balance and its interaction with the monsoon activity in East Asia. Urban planning in the monsoon Asia must consider interaction on change in the monsoon activity and urban structure and function for sustainable city in a changing climate.

## Background

Urbanization and its associated socioeconomic impacts are an essential driver of global climatic and environmental changes. Since the industrial revolution, CO_2_ emission by anthropogenic activities (i.e., fossil fuel combustion and land use change) has increased, and the amount of anthropogenic CO_2_ emissions has reached 10.7 Gt C year^−1^ over the last decade [1]. Anthropogenic CO_2_ emissions on a continent-to-country spatial scale and annual temporal scale are well known because they correlate with energy consumption data [2], and the magnitude of emissions increases exponentially with the gross domestic product [3, 4]. The ecosystem CO_2_ exchanges and surface CO_2_ balances on scales with high spatial and temporal resolution, however, involve relatively large uncertainty; thus, they hinder our understanding of the environmental and socioeconomic controlling factors of the spatiotemporal dynamics of the carbon cycle, especially in and around cities. The majority of anthropogenic activity occurs in cities, and cities are where most of the CO_2_ emission occurs. Accordingly, we require a better understanding of the carbon sources and sinks in urban areas and their spatiotemporal dynamics for our goal of a sustainable planet.

Human alteration of the Earth’s surface, such as urbanization and deforestation for food, fuel, and shelter, overwhelms the magnitude and speed of natural changes and creates an unprecedented impact on land–atmosphere interactions. Land use and cover change (LUCC) is highly involved in urbanization, which is important in local, regional, and global carbon cycles. With rapid urbanization, we are facing extensive LUCC from forest to cropland or city, and it is important that we accurately quantify changes in CO_2_ sources and sinks with LUCC in a changing climate. In a natural ecosystem, photosynthesis and respiration are key controlling processes of the carbon and surface energy balances. Vegetative canopy structure (e.g., species, density, ages, and leaf area) and physiological function (e.g., light and water use efficiencies) can explain much of the spatiotemporal variabilities of carbon sources and sinks. However, because of the complexity and heterogeneity of carbon dynamics in the urban canopy, the essential characteristics of land–atmosphere interactions can vary widely across an urbanization gradient, even under the same climatological forcing. Such complexity limits our further assessment of the carbon cycle.

With micrometeorological methods, a number of studies have been monitoring surface CO_2_ flux from various land covers over the last three decades, and currently there are more than three hundred monitoring sites across the world. Several limited studies have been conducted to compare surface CO_2_ flux along with urbanization gradient (e.g., [5–8]). Nevertheless, there is a clear gap in the measurements over the urban and suburban areas, croplands, and subtropical forests in the East Asian monsoon region, which prevents us from reducing the uncertainty of surface CO_2_ balances in the local, regional, and global carbon cycles. In particular, food and carbon securities are our concern in East Asia because of the rapid urbanization with economic growth, LUCC, and large population density that occur in addition to changes in the monsoon activity and climate. It has been reported that the East Asian monsoon plays a critical role in carbon and energy balances in the terrestrial ecosystem and energy consumption (e.g., [9–11]). Accordingly, our attention should focus on developing efficient policies toward sustainability, considering the substantial alteration of LUCC and monsoon in East Asia. In support of efforts to develop effective carbon adaptation and mitigation policies, this study will provide useful information on changes in the carbon balance in relation to LUCC due to urbanization through a comparative analysis of carbon sources/sinks under monsoon climate conditions.

This study presents eddy covariance measurement data of surface CO_2_ fluxes across an urbanization gradient in Korea where the East Asian summer monsoon affects the terrestrial ecosystem and human activity (from urban, suburban, cropland, and subtropical forest sites). In this study, we highlight the change in carbon balance due to a potential change of cropland and forest to an urban area in this critical region.

## Methods

### Surface CO_2_ balance

The surface CO_2_ balance over the urban area is given as

1 $$F_{C} + dS = C + RE - P\left( {\upmu{\text{mol m}}^{ - 2} {\text{ s}}^{ - 1} } \right)$$

where *dS*, *C*, *RE*, and *P* are the concentration change of CO_2_ in the control volume, CO_2_ emission from fossil fuel combustion, respiration by soil, vegetation, and humans, and CO_2_ uptake by photosynthesis, respectively. *dS* can be neglected by the stationary assumption of the eddy covariance method; therefore, the eddy covariance system observes *F*_*C*_, which is the sum of *C*, *RE,* and *P* in the urban area and corresponds to the net ecosystem exchange of CO_2_ (NEE). The impact of *C* is negligible at stations over natural ecosystems and cropland. The sign convention of micrometeorology is used; therefore, a positive sign indicates net CO_2_ flux from the surface to the atmosphere, and a negative sign indicates net CO_2_ uptake, presumably via photosynthesis.

### Site description

**Fig. 1:**
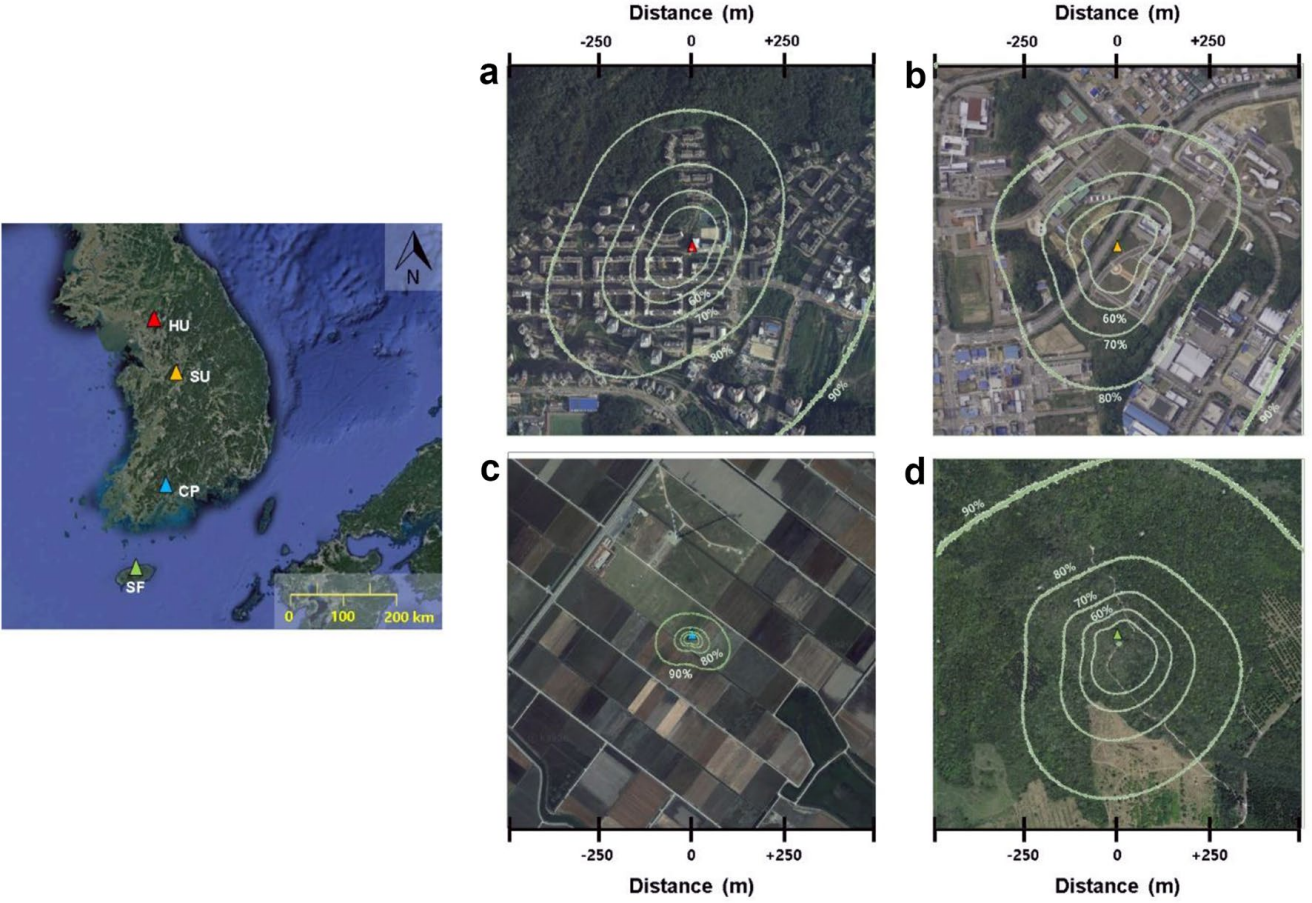
Location and footprint climatology (contour; method in Hsieh et al. [12]) under unstable conditions of study sites: **a** high-rise residential urban area (HU, red), **b** suburban area (SU, orange), **c** cropland (CP, blue), and **d** subtropical forest (SF, green)

Measurements were taken at four sites in Korea: a high-rise high-density residential area in EunPyeong, Seoul (HU: 37.6350°N, 126.9287°E; Fig. 1a); an open low-rise suburban area in Ochang, Cheongju (SU: 36.7197°N, 127.4344°E; Fig. 1b); a double-cropping rice paddy in Boseong, Jeollanam-do Province (CP: 34.7607°N, 127.2140°E; Fig. 1c); and a subtropical mixed forest on Jeju island (SF: 33.3177°N, 126.5678°E; Fig. 1d) [13]. There is an urban gradient across the sites. Buildings and roads comprised 60% and 36% of land cover at the HU and SU sites, respectively, but were negligible (< 1%) at the CP and SF sites. The mean obstacle (i.e., buildings and/or vegetation) height (z_H_) is higher at HU (~ 20 m buildings) and SF (~ 13.7 m trees) and lower at SU (~ 4 m buildings) and CP (< 1 m of crops) (Table 1). Within a 1 km radius from the SU site, there is a highway and an industrial area that generate CO_2_ emissions, which can contribute to the fluxes measured under stable atmospheric conditions. The predominant plant functional types are deciduous broadleaf trees (*Zelkova serrata*, *Cornus officinalis*, etc.) at HU, C3 grasses (*Zoysia japonica*) at SU, C3 crops (*Oryza sativa*: June–November; *Hordeum vulgare*: December–May) at CP, and deciduous broadleaf trees (*Carpinus tschonoskii*, *Quercus serrata*) at SF. Additional site information has been published previously by Hong and Hong [14] and Hong et al. [15, 16].

**Table 1:** Site characteristics and instrumentation details for the study sites

	EunPyeong (HU)	Ochang (SU)	Boseong (CP)	Jeju (SF)
Location				
Latitude (°N)	37.6350	36.7197	34.7607	33.3177
Longitude (°E)	126.9287	127.4344	127.2140	126.5678
Classification	Urban	Suburban	Rice-paddy	Mixed forest
Description	High-density high-rise residential area	Open low-rise research park	Double cropping Dec–May: *Hordeum vulgare* Jun–Nov: *Oryza sativa*	Deciduous (82%) *Carpinus tschonoskii*, *Quercus serrata*
Population density (km^−2^)	~ 15,000	770	< 50	~ 0
Building and road fraction (%)	~ 60	36	< 1	< 1
Vegetation fraction (%)	~ 40	64	~ 100	~ 100
Traffic volume (PCU day^−1^)	11,549	13,842	–	–
Measurement height (m)	30	19	2.5	27
Obstacle height (m)	~ 20	~ 4	< 1	~ 13.7
Altitude (m)	60	60	2	635
Sonic anemometer	CSAT3, Campbell Sci.	CSAT3, Campbell Sci.	CSAT3, Campbell Sci.	CSAT3, Campbell Sci.
Infrared gas analyzer	Li-7200RS, Li-COR	EC-150, Campbell Sci.	EC-150, Campbell Sci.	EC-155, Campbell Sci.
Radiometer	CNR-4, Kipp&Zonen	CNR-4, Kipp&Zonen	CNR-4, Kipp&Zonen	CNR-4, Kipp&Zonen
Data logger	CR-3000, Campbell Sci.	CR-3000, Campbell Sci.	CR-3000, Campbell Sci.	CR-3000, Campbell Sci.
Establishment	May 2013	July 2014	September 2014	May 2014
Analysis period in this study	March 2015–February 2016 (1-year)			

The 30-year mean annual precipitation at the five sites is approximately 1240 mm at the SU site, 1450 mm at the HU and CP sites, and 1920 mm at the SF site. The 30-year mean annual air temperature is approximately 12.5 °C at HU and SU, 14 °C at CP, and 17 °C at SF. During the study period, the mean annual precipitation was less than the 30-year average at the HU and SU sites but greater than the 30-year average at the CP and SF sites. More than one-half of the annual precipitation occurred during the summer (June–August) at all sites because of the summer monsoon.

### Instrumentation and data analysis

#### Measurement and data processing

The eddy covariance method was applied to monitor *F*_*C*_ at each site for 1 year from March 2015 to February 2016 (366 days) (Table 1). A 3D sonic anemometer (CSAT-3, Campbell Sci., Logan, UT) and an infrared gas analyzer (IRGA) were installed to measure the wind velocity components, sonic temperature, humidity, and CO_2_ concentration. The 10 Hz sampled data were recorded by a data logger (CR-3000, Campbell Sci., Logan, Utah.). A closed-path IRGA was used at HU (Li-7200, Li-COR, Lincoln, NE) and SF (EC-155, Campbell Sci., Logan, UT.), and an open-path IRGA (EC-150, Campbell Sci., Logan, UT.) was used at SU and CP. The 30-min averaged downward/upward short/long-wave radiation was measured by a net radiometer (CNR4, Kipp&Zonen, Netherlands).

Turbulent fluxes were computed using EddyPro software (version 6.2.0, Li-COR, Lincoln, NE) with a 30-min averaging period. Double rotation, spike removal, and spectral correction were applied with a 30-min averaging period. During the postprocessing, outliers in the 30-min CO_2_ fluxes were excluded from the data analysis based on median statistics and negative (absorption) CO_2_ fluxes during nighttime and nighttime correction is not applied [14, 17].

After quality control, the data availability was approximately 97% for HU, 52% for SU, 63% for CP, and 69% for SF. This study uses Local Standard Time (LST), which is 9 h ahead of Universal Time Coordinated (UTC).

Flux gaps were filled with an artificial neural network (ANN) using MATLAB software. For the ANN, one hidden layer with nine neurons was used with a backpropagation algorithm. The fractions of training data and independent test-set data were 80% and 20%, respectively. The variables used in the gap filling procedure were (1) hour and (2) season (fuzzy system using cosine-transformed time-of-day and day-of-year), (3) 1.5 m air temperature (T_air_), (4) 1.5 m relative humidity (RH), (5) 10 m wind speed and (6) direction, (7) downward shortwave radiation, and (8) precipitation. The meteorological variables were obtained from nearby weather observatories for each flux site: Seoul station (37.5714°N, 126.9658°E) for HU, Cheongju station (36.6392°N, 127.4407°E) for SU, Boseong-gun station (34.7633°N, 127.2123°E) for CP, and Seogwipo station (33.2461°N, 126.5653°E) for SF. All meteorological data were processed for quality control in the National Climate Data Portal (http://data.kma.go.kr/).

#### Random flux error estimation

This study evaluates the total random error (*ε*) by applying the 24-h differencing approach [18]. The 24-h differencing approach is a practical method to quantify random flux measurement error if most of the flux towers do not have two towers measuring fluxes over similar vegetation. The 24-h differencing approach calculates the random flux measurement error from measurement pairs on two successive days under the same meteorological conditions [18]. This method has been applied in various ecosystems to estimate the random error of the observed surface fluxes and has provided practical estimates of uncertainty in surface fluxes comparable to those of the sampling error model of Mann and Lenschow [19] and the two-tower approach [18, 20]. Here we will provide a brief introduction to the 24-h differencing approach; greater detail has been provided by Hollinger and Richardson [18].

If a measurement flux (*x*) pair of two successive days (*x*_*1*_ = *F* + *ε*_*1*_, *x*_*2*_ = *F* + *ε*_*2*_, where *F* and *ε* are the true flux and random error, respectively) is under equivalent meteorological conditions such as radiative flux, air temperature, humidity, and wind speed, the standard deviation of random error (*σ*(*ε*)) can be written as

2 $$\sigma \left( \varepsilon \right) \, = \sigma \left( {x_{1} - x_{2} } \right)/\sqrt 2.$$

For this 24-h differencing method, the similarity of meteorological conditions is defined for 24-h differences in photosynthetically active radiation (PAR) within 75 μmol m^−2^ s^−1^, T_air_ within 3 °C, and wind speed within 1 m s^−1^ under no-rainy condition. In addition to these filtering conditions by Richardson et al. [21], the condition of wind direction within ± 15° was added to consider the surface heterogeneity in wind direction at the sites.

## Results and discussion

### Climate conditions

**Fig. 2:**
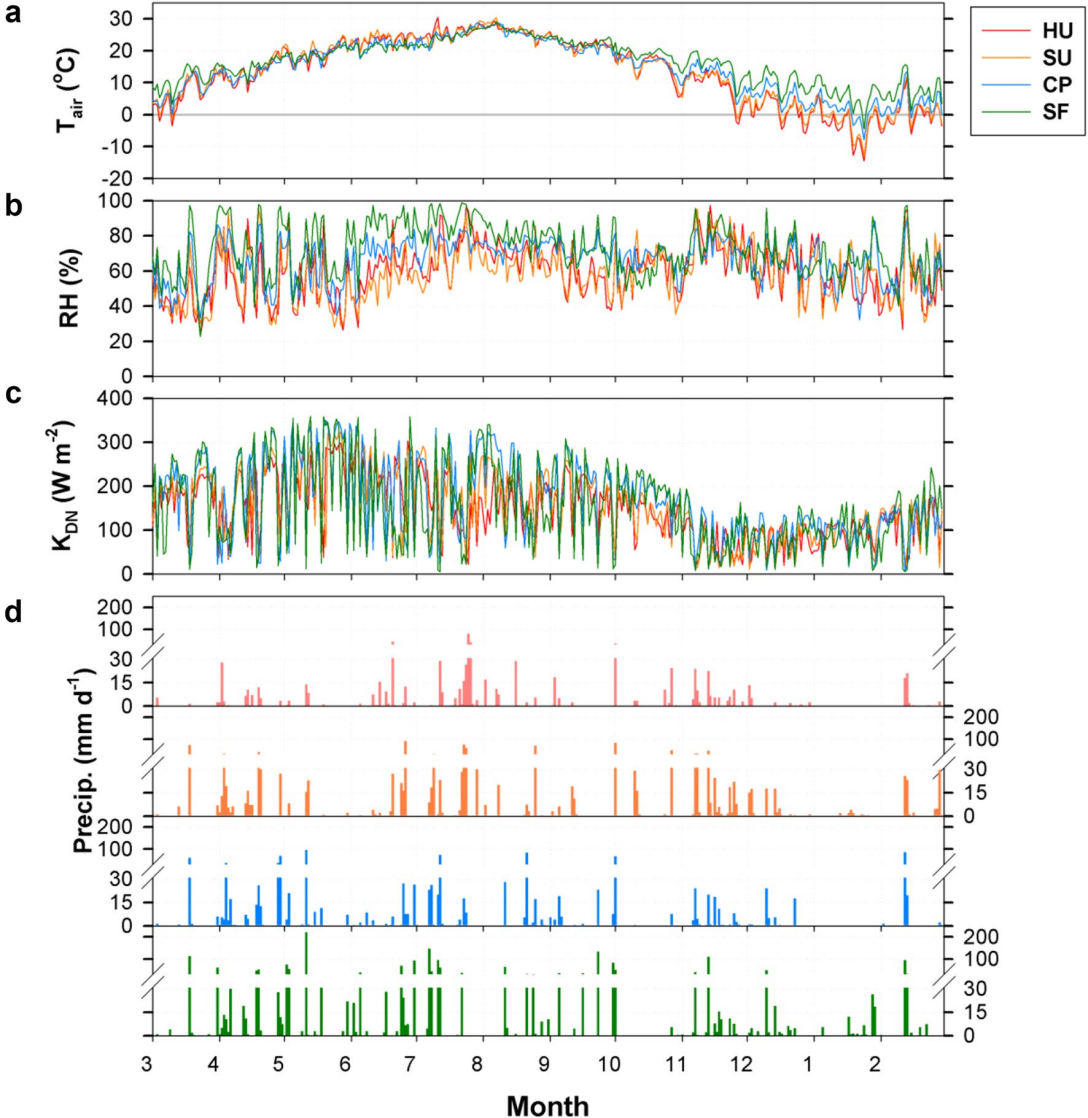
Climate conditions for March 2015–February 2016: **a** air temperature (T_air_), **b** relative humidity (RH), **c** downward shortwave radiation (K_DN_), and **d** precipitation during a year. The HU, SU, CP, and SF sites are the high-rise residential urban, suburban, cropland, and subtropical forest areas, respectively

The seasonal pattern of climate conditions is similar across the four sites with a seasonal progression of the East Asian summer monsoon (Fig. 2). The annual (March 2015–February 2016) mean T_air_ values are approximately 13.3, 13.8, 14.5, and 16.5 °C at HU, SU, CP, and SF, which differ by + 0.8, + 1.3, + 0.5, and − 0.5 °C from the 30-year average of 1981–2010, respectively. From late June to late July, Korea has “Changma,” the intense heavy rainfall period in summer, and the downward shortwave radiation decreases substantially in this period. It has been reported that this heavy rainfall period imparts a seasonal influence on the carbon and water exchanges of vegetated surfaces in East Asia (e.g., [22, 23]). With this summer monsoon influence, the summertime air temperature is similar across the sites because the same air mass affects the entire Korean Peninsula. In winter, with the retreat of the winter monsoon, there is a temperature difference between the northern sites (HU and SU) and the southern sites (CP and SF) during the study period (Fig. 2a). The annual precipitation is 807, 766, 1281, and 2575 mm year^−1^ (56%, 62%, 88%, and 134% of the 30-year average) for HU, SU, CP, and SF, respectively, but the timing of rainfall events is similar across the sites. The precipitation differences among the sites are related to the amount of rainfall in the same summer rain events rather than the timing of the rainfall events.

### Flux measurement uncertainty

Micrometeorological measurements have several sources of error, including both random and systematic errors that can lead to flux uncertainties. Systematic errors can often be quantified and corrected by regular sensor calibration. Random errors, however, can grow larger through error propagation and must be quantified in order to conduct a proper data interpretation and model evaluation. We assess the systematic uncertainties in CO_2_ fluxes calculated by the different data processing methods. Individual data processing procedures produce nonnegligible uncertainties of approximately 5% of CO_2_ fluxes (Table 2). CO_2_ fluxes have relatively high sensitivity to detrending methods such as planar fit rotation, outlier removal, and a high-pass filter.

**Table 2:** CO_2_ fluxes and their uncertainties with different processing procedures

*F*_*C*_ (unit: μmol m^−^^2^ s^−^^1^)	Mean	Std.	25%	50%	75%
Default (*double rotation & block averaging* only)	8.8	7.3	2.3	7.8	14.4

Difference with default (EXP-default; unit: μmol m^−2^ s^−1^)					
Coordinate rotation					
Triple rotation	− 0.1	1.5	0.0	0.0	0.1
Planar fit [24]	− 0.8	3.8	− 1.7	− 0.5	0.3
Planar fit [25]	− 1.0	3.9	− 1.9	− 0.6	0.3
Detrending					
Linear detrending	− 0.4	3.9	− 1.3	− 0.1	0.4
Running mean 250 s	− 0.7	4.3	− 2.0	− 0.5	0.8
Exponential running mean 250 s	− 0.9	3.8	− 2.0	− 0.6	0.2
Time Lags Compensation					
Covariance maximization	0.0	0.1	0.0	0.0	0.1
*Covariance maximization with default*	0.1	0.1	0.0	0.0	0.1
Automatic time lag Optimization	0.0	0.1	0.0	0.0	0.1
Spike count/removal					
Mauder et al. [26], accepted spikes 1–30%	− 0.5	1.4	− 0.3	0.0	0.0
*Vickers and Mahrt* [27] *(VM97)*, *Max consecutive outliers (MCO) 3*	− 0.1	0.2	− 0.1	0.0	0.0
VM97, MCO 10	− 0.2	0.4	− 0.2	0.0	0.0
VM97, MCO 30	− 0.3	0.7	− 0.4	− 0.1	0.0
VM97, MCO 50	− 0.4	0.8	− 0.4	− 0.1	0.0
VM97, MCO 70	− 0.4	0.8	− 0.5	− 0.1	0.0
Amplitude resolution					
4–7 sigma, number of bins 50–150	0.0	0.1	0.0	0.0	0.1
Dropouts					
Accepted central dropouts 5–20%	0.0	0.1	0.0	0.0	0.1
Absolute limits	0.0	0.1	0.0	0.0	0.1
Skewness & Kurtosis	0.0	0.1	0.0	0.0	0.1
Discontinuities	0.0	0.1	0.0	0.0	0.1
Spectral correction					
*Low*-*frequency range* [28]	0.3	0.4	0.1	0.2	0.4
High-frequency range (HFR) [29]	0.1	0.2	0.0	0.1	0.2
HFR [30, 31]	0.7	1.0	0.2	0.4	0.8
HFR [32]	0.2	0.3	0.0	0.1	0.4
HFR [33] with Horst and Lenschow [34] (HL09): along-wind, cross, and vertical winds	0.1	0.2	0.0	0.1	0.2
HFR [33] with HL09: only cross and vertical winds	0.1	0.1	0.0	0.1	0.2
HFR [35] with HL09: along-wind, cross, and vertical winds	0.2	0.2	0.0	0.1	0.3
*HFR* [35] *with HL09: only cross and vertical winds*	0.2	0.2	0.0	0.1	0.3

Data period is 1-month (March 2015) from HU site. Italic indicates the methods applied in this study

**Fig. 3:**
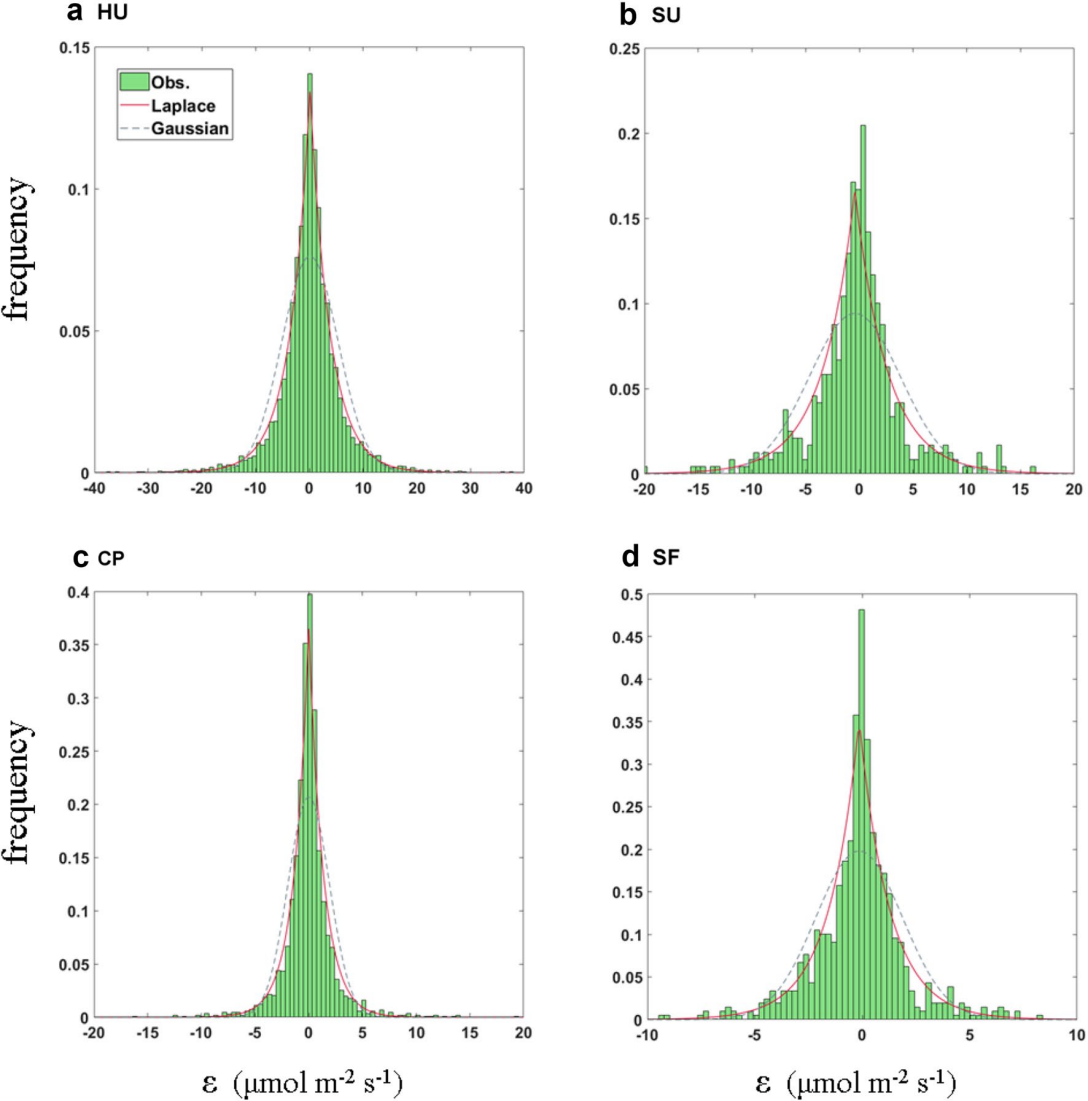
Probability distribution of random flux error (ε). The HU (**a**), SU (**b**), CP (**c**), and SF (**d**) sites are high-rise residential urban, suburban, cropland, and subtropical forest areas, respectively. The red lines and blue dashed lines are Laplace distributions and Gaussian distributions, respectively

Several general statistical characteristics are robust in the probability density function (PDF) of the random error (*ε*) of CO_2_ flux across the gradient of urbanization and ecosystem types (Fig. 3). First, the probability distribution is symmetrical around the average value with peaky maximum and heavy tails. The Kolmogorov–Smirnov test rejects the hypothesis that the PDF has a Gaussian distribution (*p *< 0.01) and the Laplace (double exponential) distribution is a better approximation than the Gaussian distribution. Indeed, the skewness and kurtosis are large and positive for all sites, such that the PDF deviates significantly from the Gaussian distribution, and they have particularly large values during the nighttime. Our findings are similar to those of previous studies of forest and grass canopies (e.g., [18, 21]).

**Fig. 4:**
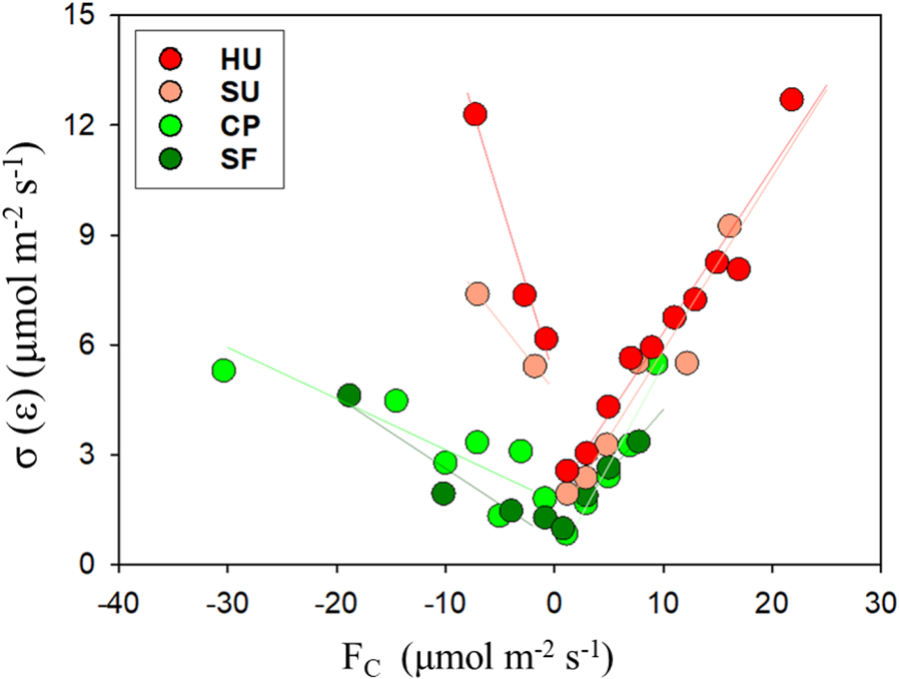
Net CO_2_ fluxes and standard deviation of random flux error. The HU, SU, CP, and SF sites are high-rise residential urban, suburban, cropland, and subtropical forest areas, respectively. Colored lines come from the linear regression of the data at the sites in Table 3

Second, random flux uncertainty has different variability with turbulent fluxes (i.e., heteroscedasticity) (Fig. 4). The standard deviation of the random error is proportional to the magnitude of the CO_2_ flux ($\left| {F_{C} } \right|$) in all the sites, as calculated via Eq. (3):

3 $$\sigma \left( \varepsilon \right) = a + b \left| {Fc} \right| .$$

The intercept, *a*, ranges from − 0.02 μmol m^−2^ s^−1^ for the CP site to 1.83 μmol m^−2^ s^−1^ for the HU site. The slope, *b*, varies between 0.34 and 0.55 across the sites (Table 3). The slope is larger in positive (nighttime) than negative (daytime) CO_2_ fluxes in forest and cropland. In contrast, the opposite is true for the urban and suburban sites (i.e., HU and SU). Richardson et al. [20] attributed a larger slope in the daytime compared with nighttime to either data editing or different turbulent transport statistics during the day and night. Positive CO_2_ fluxes are, however, possible in the daytime at the HU and SU sites because of the predominance of anthropogenic CO_2_ sources. In the case of the HU site, the uncertainty estimation shows similar characteristics even after considering different human activities between weekends and weekdays (not shown here).

**Table 3:** The linear relationship between random flux error and the corresponding flux magnitude

Site	F_C_ ≤ 0	F_C_ ≥ 0
HU	− 0.97 × F_C_ + 5.11 (0.99)	0.45 × F_C_ + 1.83 (0.95)
SU	− 0.37 × F_C_ + 4.75 (1.00)	0.48 × F_C_ + 1.11 (0.92)
CP	− 0.14 × F_C_ + 1.74 (0.69)	0.55 × F_C_ − 0.02 (0.95)
SF	− 0.20 × F_C_ + 0.67 (0.89)	0.34 × F_C_ + 0.82 (0.99)

The number in the parentheses indicates the correlation coefficient

In general, our findings are consistent with those of previous studies and indicate that a constant relative error is inappropriate for parameter optimization and data assimilation [21]. The PDFs of random error in the urban and suburban stations also share similar characteristics with natural vegetation canopies. However, the slope and intercept are larger for the urban-influenced stations (i.e., HU and SU) than for cropland and forest (i.e., CP and SF), indicating that random flux uncertainties are much larger in an urban area than in natural forest or cropland. Although our estimation was generated by dividing wind direction into narrow ranges, the surface heterogeneity of urban structure and function and the larger relative error will have potential impacts on random error variability. Another plausible explanation for this larger relative random error is that anthropogenic activity is not mainly controlled by meteorological conditions, thus invalidating the 24-h differencing approach in the HU site.

### Temporal dynamics of CO_2_ flux along the urbanization gradient

**Fig. 5:**
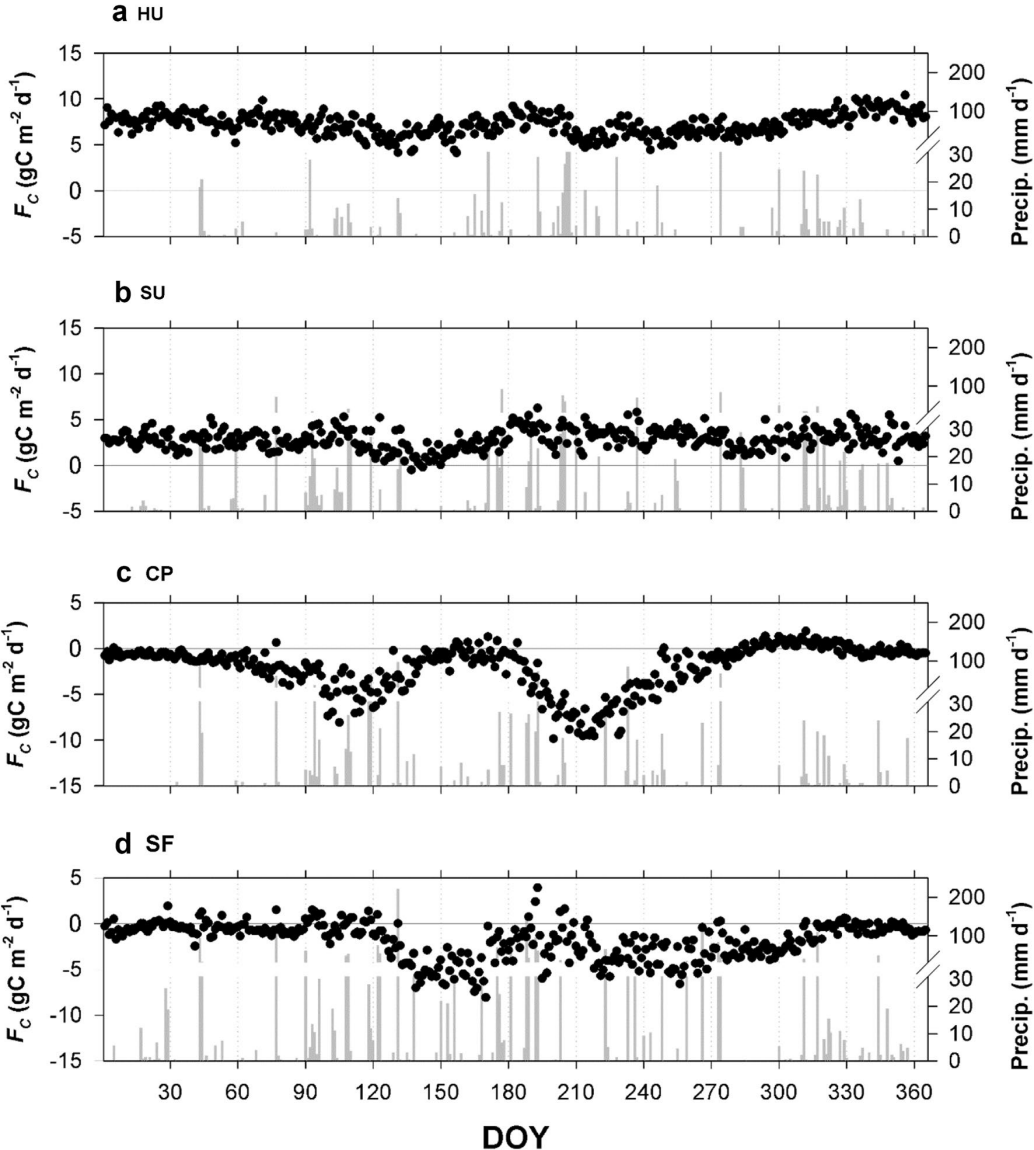
Daily CO_2_ fluxes and precipitation at **a** high-rise residential urban (HU), **b** suburban (SU), **c** cropland (CP), and **d** subtropical forest (SF) sites from March 2015 to February 2016

**Fig. 6:**
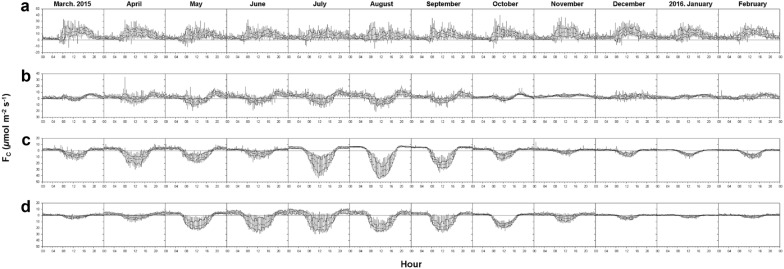
Monthly mean diurnal pattern of CO_2_ flux during 1 year (March 2015–February 2016) at the four sites with median, interquartile range (box), and 5th and 95th values (whiskers): **a** high-rise residential urban area (HU), **b** suburban area (SU), **c** cropland (CP), and **d** subtropical forest (SF)

There are distinct differences in net CO_2_ fluxes among the sites across the gradient of urbanization from the perspectives of diurnal and seasonal variations of net CO_2_ exchange (Figs. 5 and 6). The SF site is a strong CO_2_ sink from the end of April through October, and the maximum CO_2_ absorption rate is − 25 μmol m^−2^ s^−1^ during this period (Figs. 5d and 6d). Importantly, during the summer growing season, the SF site shows an obvious mid-season decline of carbon uptake with a substantial reduction in the solar radiation. In other words, the forest site exhibits strong carbon uptake after the leaf-out in early May, which significantly decreases with the onset of the summer monsoon and regains its strong carbon uptake on non-rainy summer days. The carbon uptake in the forest canopy continues until defoliation in late October. Such a bimodal peak of NEE is a typical seasonal variation in forest canopies that is influenced by the Asian summer monsoon (Figs. 5d and 6d) (e.g., [9, 22]).

The cropland also shows a bimodal peak of carbon uptake but in a different period from the forest (from April to August) because of a double-cropping farming system (planting and subsequent harvest of barley and rice). NEE shows rapid changes in sign during the harvest of crops in June (barley) and November (rice) at the CP site. The maximum CO_2_ absorption rate is approximately − 40 μmol m^−2^ s^−1^ for rice in July–August, which is comparable to previous results from rice paddies in East Asian countries (e.g., [36–40]). The maximum CO_2_ absorption rate of barley is smaller (approximately − 20 μmol m^−2^ s^−1^) during the mid-growing period of barley (from April to May).

Unlike the forest and cropland, the HU and suburban (SU) areas are sources of CO_2_ to the atmosphere throughout the year, and the seasonal variability of CO_2_ flux is relatively small compared with the cropland and forest. Nonetheless, we can see the influence of the heavy rain period in summer, possibly because of urban vegetated surfaces at both the urban-influenced sites (Fig. 5). Indeed, the suburban area exhibits daytime carbon uptake (i.e., negative CO_2_ flux) in the summer growing season (from March to October). The urban site does not show negative fluxes even in the summer, but the positive CO_2_ fluxes do decrease in a similar manner as at the suburban site in the summer.

Figure 6 shows the diurnal variation of CO_2_ flux. Two peaks of CO_2_ flux are evident, during the morning and evening rush hours at HU. However, in the suburban area, vegetated surfaces in the flux footprint offset the afternoon rush hour effect, and the maximum carbon uptake occurs around noon when the solar radiation is at its maximum. Traffic volumes at HU and SU show the similar diurnal pattern with rush hour peaks and SU has larger traffic volume than HU (Table 1). Consequently, the maximum CO_2_ emission rate of the SU site is half that at the HU site (approximately 10 μmol m^−2^ s^−1^), and its timing is delayed to the late evening despite the larger traffic volume at SU. It is also notable that these two peaks in the rush hours become smaller during the summer season, indicating that vegetated surfaces mitigate anthropogenic CO_2_ emissions in the summer growing season. The seasonal variation of anthropogenic CO_2_ emission at the HU and SU sites are relatively small possibly because of the district (HU site) and electricity (SU site) heating system not to make anthropogenic CO_2_ emission. In this respect, the seasonal course of CO_2_ flux shows a mid-season depression of CO_2_ absorption, with two minima around May and September in the urban and suburban areas, with the combination of vegetative uptake and the lengthy summer monsoon period as the vegetative carbon uptake decreases in July during heavy summer rain spells. Previous studies reported that such a mid-season depression is related to the effect of heavy rain spells on ecosystem function in the Asian summer monsoon season (e.g., [9, 23]). Our result suggests that the interplay of urban vegetation and summer monsoon activity and CO_2_ flux in cities in the East Asian monsoon region should also be interpreted with the seasonal progression of the East Asian monsoon similarly to natural vegetation in this region.

### Light use efficiency of CO_2_ fluxes

**Fig. 7:**
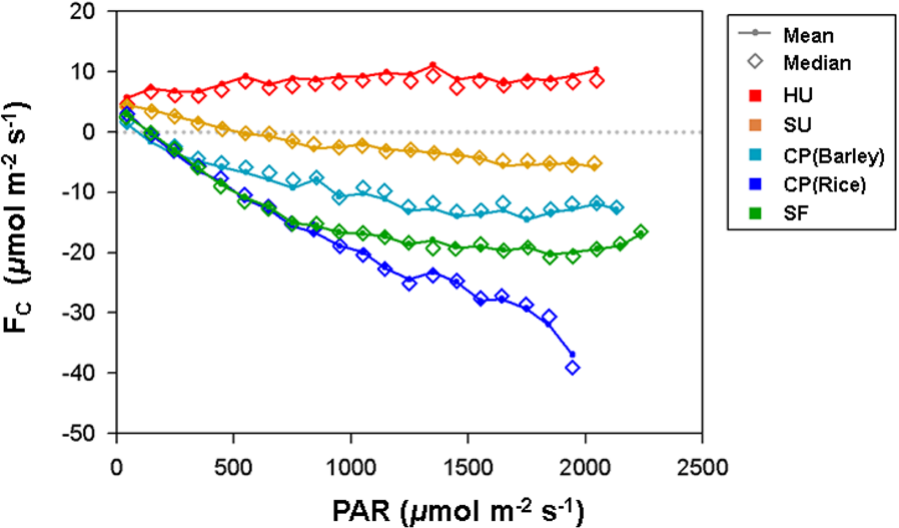
Light-response curve for the growing season: May–September for high-rise residential urban (HU), suburban (SU), and forest sites (SF); April–May for barley and July–September for rice at the cropland site (CP)

Figure 7 shows the light-response curve during the growing season: May–September for urban, suburban, and forest sites; and April–May for barley and July–September for rice at the cropland site. All the sites except for HU tend to increase CO_2_ uptake from the atmosphere (i.e., negative *F*_*c*_) as PAR increases. The cropland and forest sites show increases in carbon uptake with increasing PAR that are similar to previous reports for various vegetative canopies (e.g., [41, 42]). In the rice paddy, the photosynthesis rate continues to increase as PAR intensifies without the light saturation, thus leading to larger light use efficiency (LUE) compared with the forest. We speculate that this large LUE is related to the ample nutrients and water supplied to the rice paddy by fertilization and irrigation.

The suburban site shows the typical pattern of vegetative canopies as LUE increases. However, in the SU site, a positive *F*_*c*_ is maintained up to a relatively large PAR of approximately 500 μmol m^−2^ s^−1^, as compared with other natural canopies. This suggests that the sign change of *F*_*c*_ from positive to negative with higher PAR is related to the compensation of CO_2_ emission by vegetation around the tower. The net CO_2_ emission rate at the high-rise residential site does not change significantly regardless of PAR variation and shows two peaks during the rush hours corresponding to approximately 600 and 1300 μmol m^−2^ s^−1^ in PAR. These results indicate the predominance of carbon emission from cars, which does not depend on temperature. It is also noticeable that CO_2_ fluxes with small PAR are larger at the urban-influenced sites (HU and SU) than at those with vegetative canopies (CP and SF), but they are much smaller than in other cities reported by Ward et al. [7].

### Temperature responses of CO_2_ flux

**Fig. 8:**
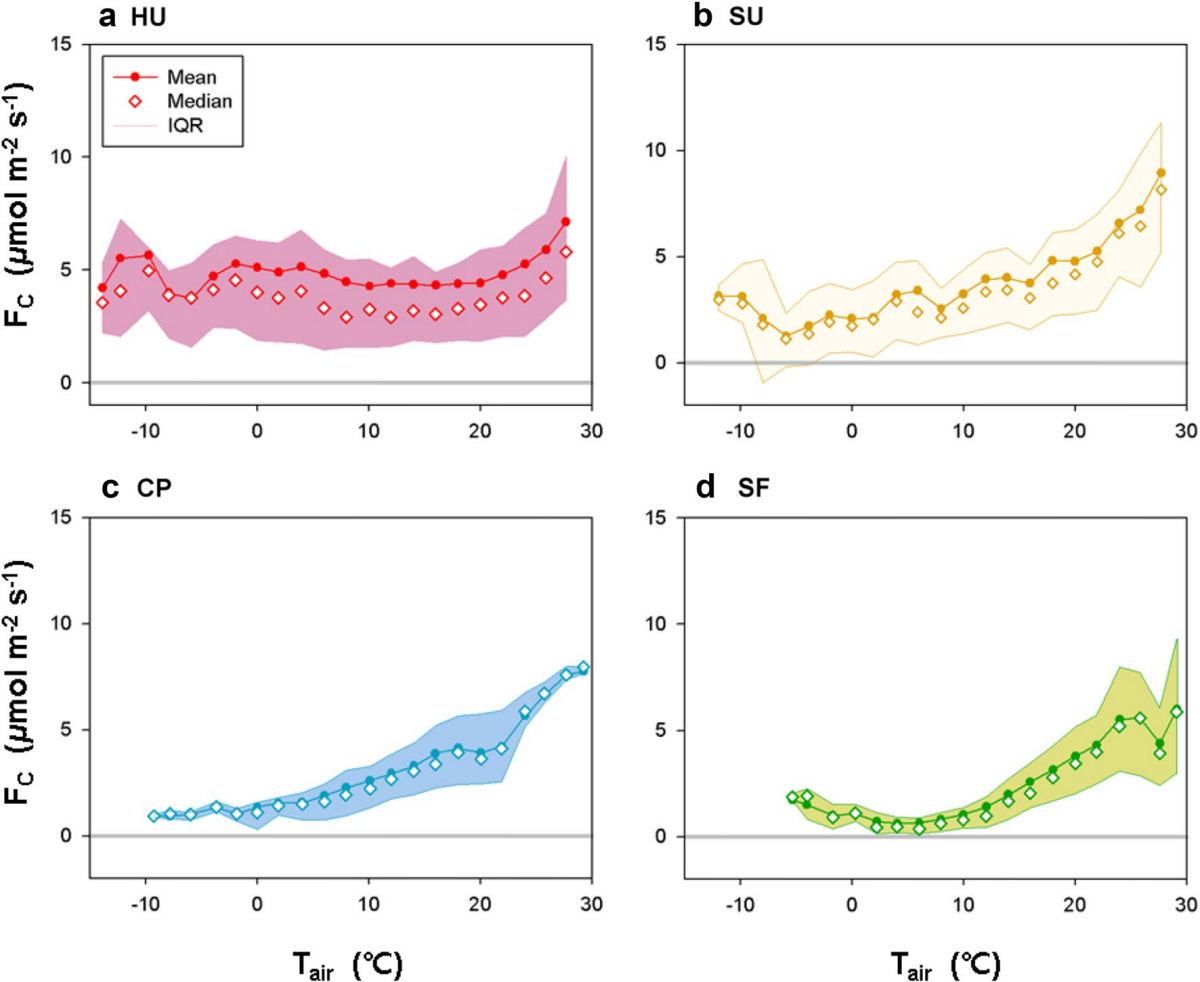
Temperature-response curve for nocturnal CO_2_ flux during one-year (March 2015–February 2016) at the four sites with mean, median, and interquartile range (IQR): **a** high-rise residential urban area (HU), **b** suburban area (SU), **c** cropland (CP), and **d** subtropical forest (SF)

Figure 8 is a nighttime temperature-response curve of CO_2_ flux. It has been reported that in natural ecosystems, nighttime CO_2_ flux is an exponential function of T_air_ because warmer temperature creates favorable conditions for ecosystem respiration [43, 44]. Our results also show this typical dependency of nocturnal *F*_*c*_ on T_air_ except for the HU area. The HU area exhibits the typical temperature dependence only in the summer season (> 20 °C range) and shows nearly constant CO_2_ flux with changes in T_air_ indicating possible contribution of ecosystem respiration in summer at the urban residential area.

**Fig. 9:**
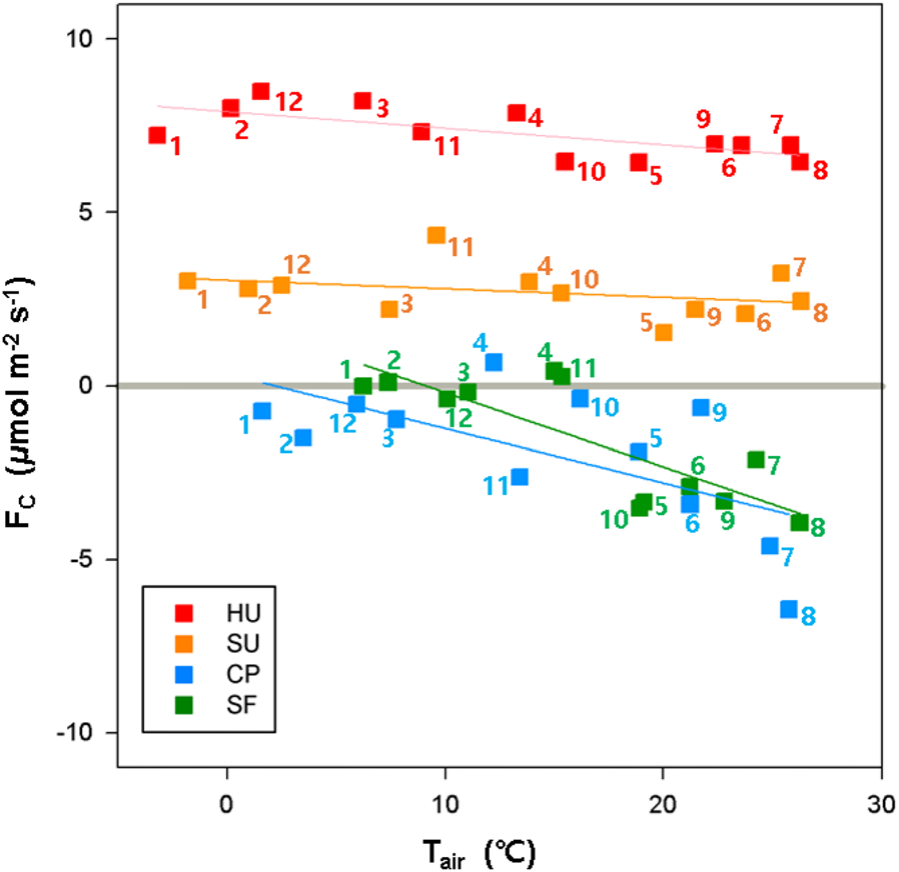
Relationship between monthly mean CO_2_ flux (F_C_) and monthly mean air temperature (T_air_) for 1 year (March 2015–February 2016) at the four sites. Numbers (1–12) indicate the corresponding month. The HU, SU, CP, and SF sites are high-rise residential urban, suburban, cropland, and subtropical forest areas, respectively

Net carbon emission is nearly constant throughout the year at the HU and SU sites, and CO_2_ emissions do not show significant variations with changes in T_air_ (Fig. 9). It is mainly because of (1) the power plant is located out of the source area and (2) both sites are using the heating system without CO_2_ emissions, leading to nearly constant anthropogenic activities. Compared with the crop and forest canopies, the temperature-response curve for nocturnal CO_2_ flux has a relatively wider range at the urban and suburban sites, and the mean values are larger than the medians with increases in urbanization (i.e., positive skewness) (Fig. 8). Anthropogenic emissions exhibit more asymmetry in their diurnal trends than emissions from plants and soils because anthropogenic activities such as heating and transportation tend to be higher in the afternoon than in the morning, given the air temperature at the HU and SU sites (Fig. 6), suggesting that the observed CO_2_ fluxes at the HU and SU sites reflect strong anthropogenic CO_2_. The baseline of the *F*_*c*_ − T_air_ relationship at the HU site is larger than at the other three sites (approximately 5 μmol m^−2^ s^−1^) but smaller than in other cities (about 50 μmol m^−2^ s^−1^ in city center of London, about 10 μmol m^−2^ s^−1^ in the Swindon suburban site [7]; and about 20 μmol m^−2^ s^−1^ in Beijing, China [45]).

CP and SF sites show a larger spread of the distribution of CO_2_ flux with warm air temperatures (15–20 °C for CP and above 20 °C for SF). For the forest canopy (SF), warm climate conditions of > 20 °C correspond to the summer growing season (May–October) and include the heavy rain spell, Changma (late June–late July). This result shows that ecosystem respiration in summer is sensitive to the timing and duration of rainfall events during the Changma, creating larger variability through substantial variations of the downward shortwave radiation and surface moisture in this region [22, 23]. On the contrary, it is not obvious that the seasonal progression of the monsoon makes such an impact on the crops, probably because of the human management of the rice paddy. Instead, it is observed that abrupt changes in vegetative surfaces occur during the harvest of barley and during the planting (May) and harvest (October) of rice, and such periods match up with large uncertainties in ecosystem respiration in the range of 15–20 °C (Fig. 8c).

Monthly average CO_2_ fluxes produce negative relationships because of the carbon uptake in summer (Fig. 9). It is notable that the HU (− 0.05 μmol m^−2^ s^−1^ °C^−1^) and SU (− 0.02 μmol m^−2^ s^−1^ °C^−1^) sites produce a less steep negative slope between T_air_ and *F*_*c*_ compared with other cities in previous studies (− 0.56 and − 1.95 μmol m^−2^ s^−1^ °C^−1^ at Swindon and London, UK [7]; − 0.34 μmol m^−2^ s^−1^ °C^−1^ in Beijing, China [45]; − 0.25 μmol m^−2^ s^−1^ °C^−1^ in Tokyo, Japan [46], 2004; and − 0.2 μmol m^−2^ s^−1^ °C^−1^ in Łódź, Poland [47]). Around the HU site, the high-rise residential buildings use a district heating system, which uses hot water coming through pipes from remote power plants. In contrast, the houses and buildings around the SU site usually use electricity for heating. In addition, the resident population is small (< 700 people km^−2^), and the vegetated surface mitigates fossil fuel emissions through photosynthesis. Consequently, these societal environments around the HU and SU stations do not contribute to the local CO_2_ emissions, thus producing a relatively weak negative correlation compared with the previous studies. In contrast, there is a strong seasonal variation in CO_2_ fluxes at the cropland and forest canopy sites, with the seasonality of vegetation shown in the light-response curve (Fig. 7).

### Annual net CO_2_ fluxes

**Fig. 10:**
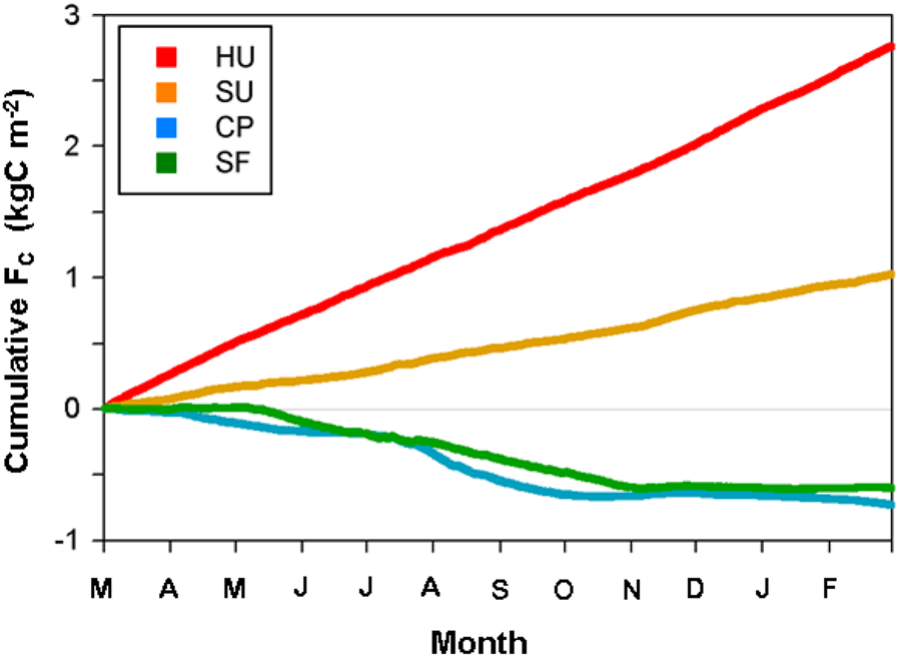
Cumulative CO_2_ fluxes (F_C_) during 1 year (March 2015–February 2016) for four sites. The HU, SU, CP, and SF sites are high-rise residential urban, suburban, cropland, and subtropical forest areas, respectively

The annual net CO_2_ fluxes are 2.75, 1.02, − 0.73, and − 0.60 kg C m^−2^ year^−1^ for the urban, suburban, cropland, and forest sites, respectively. The urban and suburban sites are carbon sources to the atmosphere throughout the year and show monotonically increasing cumulative CO_2_ fluxes because of the weak seasonality (Fig. 10).

**Fig. 11:**
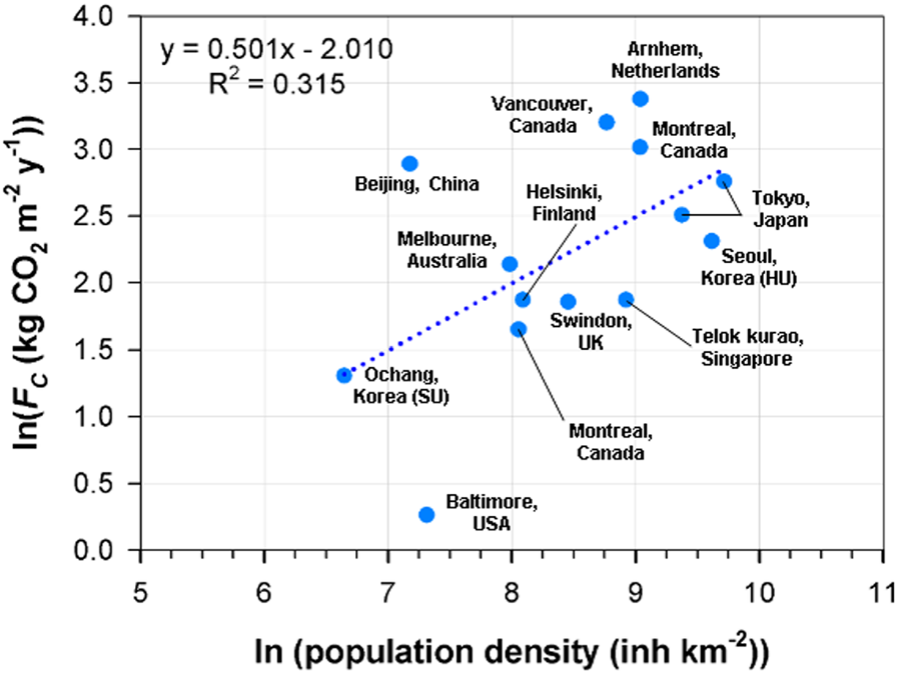
Annual net CO_2_ fluxes (*F*_*C*_) and population density in suburban and residential areas. HU and SU are the high-rise residential urban and suburban areas in this study, and the other values come from Ward et al. [7], Kleingeld et al. [48], and Björkegren and Grimmond [49]. The blue dotted line represents a linear regression

Figure 11 shows CO_2_ fluxes in suburban and residential areas vs. population density. In general, net CO_2_ flux in a suburban and urban residential area has sublinear scaling with population density (*P*; number of inhabitants per km^2^, inh km^−2^) on a logarithmic scale:

4 $$Fc = Fc_{0} \cdot P^{\alpha }$$

where $Fc_{0}$ is net CO_2_ flux at a zero population of 0.13 kg CO_2_ m^−2^ year^−1^ and *α* is the scaling exponent of 0.50 from the linear regression with *R *= 0.56. In other words, the relationship exhibits sublinear scaling (*α* < 1), and therefore a larger urban population density is more efficient with regard to net CO_2_ flux. However, this sublinear relationship has a smaller correlation coefficient (i.e., a wide spread of CO_2_ flux at the given population density) and a less steep slope compared with inventory data analysis. For example, Fragkias et al. [50] reported a slope of 0.93 with *R *= 0.99 in US cities, and the top 500 CO_2_-emitting cities reported in Moran et al. [51] had a slope of 0.72 with *R *= 0.93 (calculated in this study based on their data). Anthropogenic CO_2_ emission around HU and SU are 6.0 and 3.3 kg C m^−2^ year^−1^ from the ODIAC emission dataset [52]. If we consider that our measurement includes both fossil fuel emissions and vegetative carbon uptake, our result suggests that urban vegetation is important to offset anthropogenic emissions in urban areas; thus, the net CO_2_ flux for cities will depend on population density, traffic volume, and vegetation cover fraction.

**Fig. 12:**
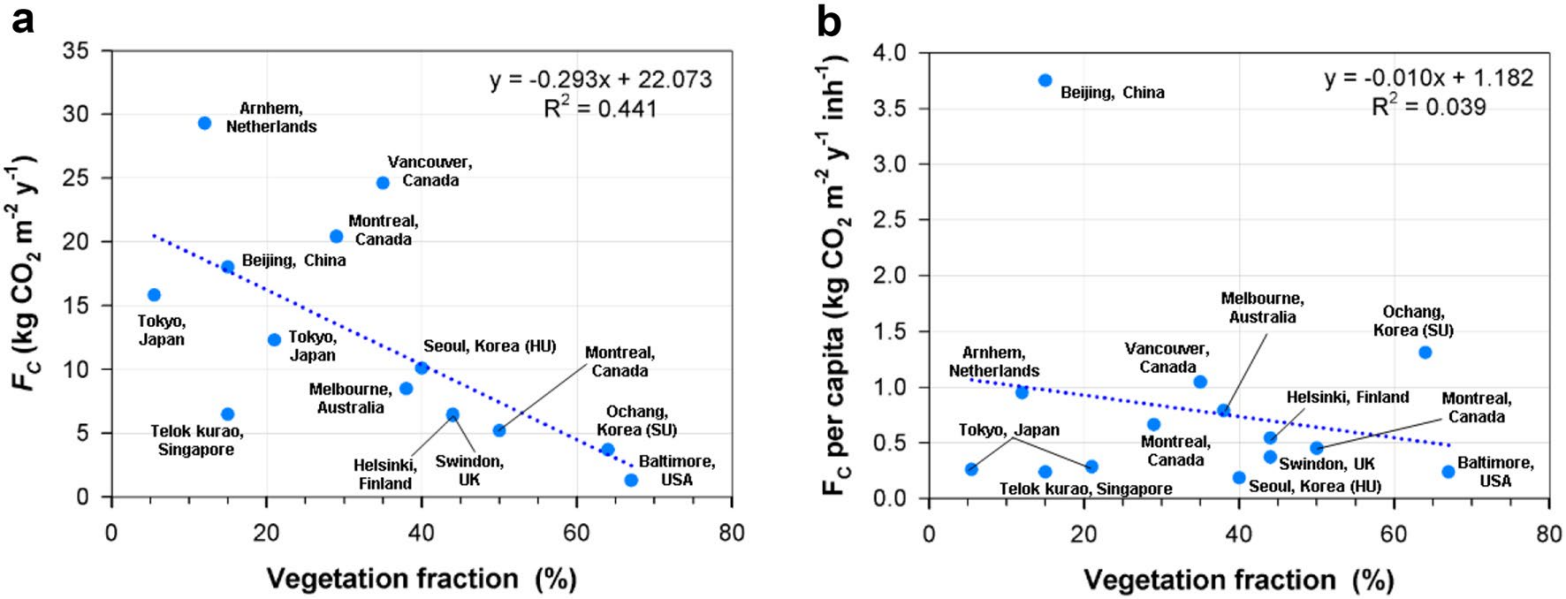
Relationship between **a** annual CO_2_ flux and **b** annual CO_2_ flux per capita as a function of vegetation fraction (previous study results are derived from Ward et al. [7], Kleingeld et al. [48], and Björkegren and Grimmond [49]). The HU and SU sites are high-rise residential urban and suburban, respectively

Indeed, the net CO_2_ emissions per capita at the urban (HU) and suburban (SU) sites were 0.7 and 4.9 t CO_2_ year^−1^ person^−1^, respectively, and they scale with population density similarly to other cities (Fig. 11, Table 4). The HU site shows a smaller CO_2_ emission rate than Tokyo, Japan, which has a similar population density and vegetation fraction [46, 55]. It is notable that the vegetation cover can partially explain the outliers from this subscaling (Fig. 12). The smaller CO_2_ emission rate at HU can be attributed to the larger vegetation fraction compared with Tokyo, Japan. The higher CO_2_ emission rate per capita in Beijing, China also corresponds to a smaller vegetation fraction (< 20%). It is noticeable that the large CO_2_ emission rates per capita (> 0.65 t C year^−1^ inh^−1^) in Vancouver and Montreal, Canada [6, 56] and Arnhem, Netherlands [48] are scaled well with changes in vegetation fraction but are much stronger than other cities. We speculate that major CO_2_ sources of these sites are from space-heating systems and low vegetation fraction (Fig. 12b).

**Table 4:** Annual net CO_2_ fluxes (*Fc*) from suburban and urban residential areas in the literature

Location	Vegetation fraction, *Fv* (%)	Population density, *P* (inh km^−2^)	*Fc* (kg CO_2_ m^−2^ year^−1^)	*Fc/P* (t C year^−1^ inh^−1^)	References
Seoul, Korea	40	15,000	10.1	0.18	This study
Ochang, Korea	64	770	3.7	1.31	This study
Tokyo, Japan	21	11,800	12.3	0.28	[52]
Tokyo, Japan	5.5^a^	16,600	15.8	0.26	[55]
Beijing, China	15	1309	18.0	3.75	[58]
Singapore	15	7491	6.5	0.24	[53]
Melbourne, Australia	38	2939	8.5	0.79	[5]
Helsinki, Finland	44	3262	6.5	0.54	[54]
Arnhem, Netherlands	12	8425	29.3	0.95	[48]
Swindon, UK	44	4700	6.4	0.37	[7]
Vancouver, Canada	35	6420	24.6	1.05	[56]
Montreal, Canada (URB)	29	8400	20.4	0.66	[6]
Montreal, Canada (SUB)	50	3150	5.2	0.45	
Baltimore, USA	67	1500	1.3	0.24	[61]

^a^Vegetation fractions in summer and winter are equally averaged

The CO_2_ emission rate in HU site is also smaller than the inventory values (about 12 t CO_2_ year^−1^ inh^−1^) on the regional scale around the Seoul metropolitan area [51, 57]. It has been reported that typical uncertainties of the inventory-based emission estimates are smaller (< 10%) in national to global scales but larger (< 30%) in city scale [58–61]. In particular, the anthropogenic emission has large uncertainties at the outskirt of the city such as the HU and SU sites [60]. The smaller observed CO_2_ emission rate is also attributable to the spatio-temporal mismatch of heating system, traffic amount, and vegetation activities between the inventory-based emission estimates and in situ flux measurements. Indeed, the power plant contribution is not in the flux footprint unlikely to the area wide inventory-based emission estimates.

The net CO_2_ emission at the SU site obeys the scaling relationship given by Eq. (4) but shows larger CO_2_ emission than Baltimore, USA, which has a similar vegetation fraction (> 0.5) but a larger population density [62]. In addition, despite the smaller population density and the larger vegetation fraction around the SU site, the traffic volume around the SU site is larger than around the HU site; therefore, heavy traffic volumes and factories in the suburban area contribute to additional CO_2_ emissions into the atmosphere. Because urban CO_2_ emissions can be interpreted by a measure of energy consumption and traffic volume, it is likely that the district heating system around the HU site contributes a much smaller net CO_2_ emission compared with the SU site. This finding is consistent with the results of Makido et al. [63], who reported less CO_2_ emission from the passenger transportation sector from compact cities compared with more sprawling cities and a less steep slope of *F*_*c*_ to T_air_ in the urban and suburban areas (Fig. 9).

**Fig. 13:**
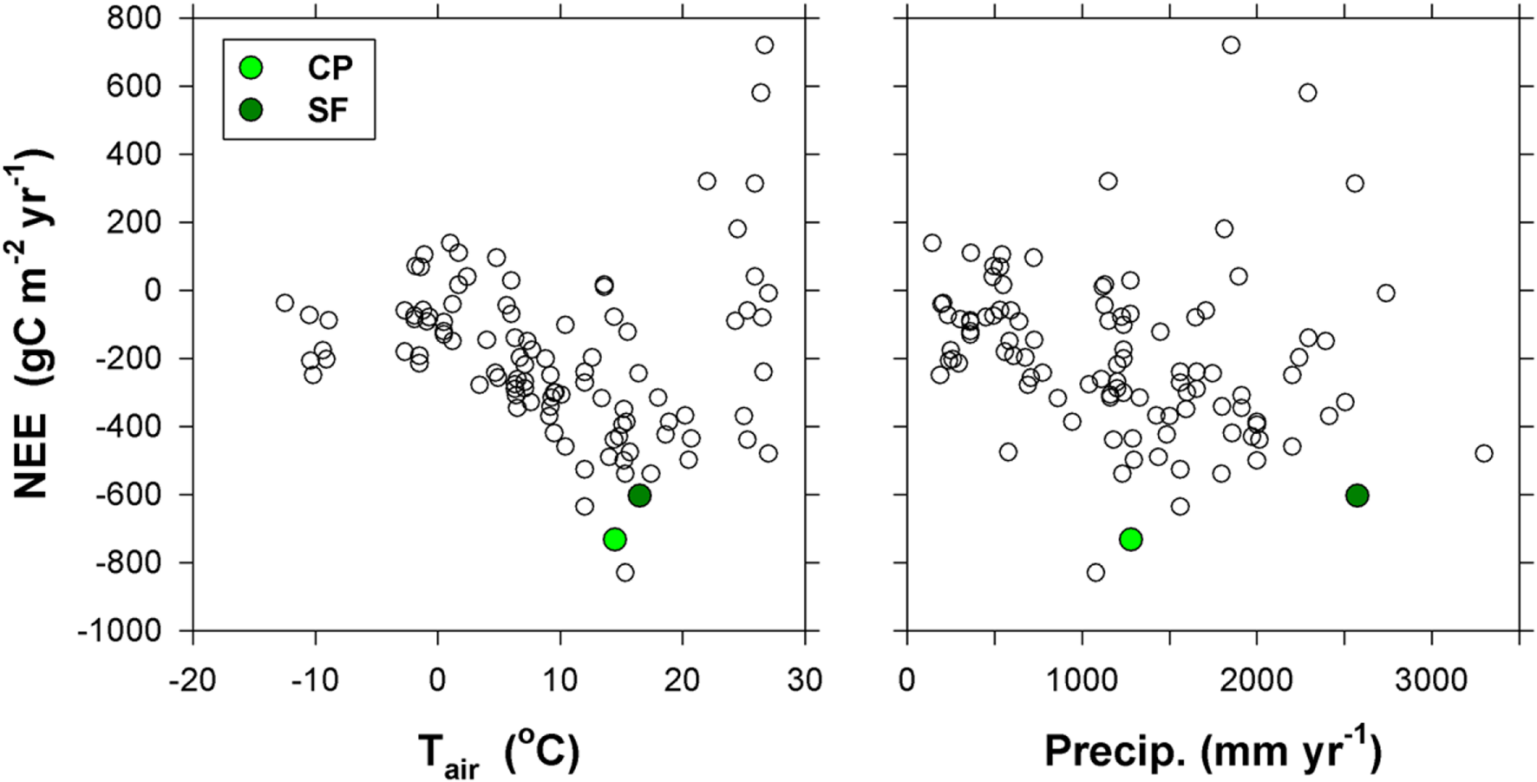
Relation between annual net ecosystem exchange of CO_2_ (NEE) with mean annual air temperature (T_air_, left) and precipitation (right) from March 2015 to February 2016. The values of previous studies are derived from Valentini et al. [62], Curtis et al. [63], Kato and Tang [64], Hirata et al. [65], and Takagi et al. [66]. The CP and SF sites are cropland and subtropical forest, respectively

The annual mean CO_2_ fluxes of the four sites and the controlling factors are comparable to those of previous studies: e.g., traffic volume and vegetation fraction in urban areas [7, 8, 63] and air temperature and precipitation in crops and natural vegetation canopies in various climate zones [64–68] (Figs. 12 and 13). Our results for the HU and SU sites are also within the range of the relationship between carbon emissions and vegetation fraction reported by Ward et al. [7] and Lietzke et al. [69] (Fig. 12).

The CP and SF sites are strong carbon sinks compared with other vegetation canopies (Fig. 13). In particular, the CO_2_ uptake of the cropland is large because the double-cropping system and human management alleviate the effects of the heavy rain spells in summer, thus producing larger carbon uptakes in spring and summer with T_air_ > 10 °C. The slopes of carbon uptake accumulation corroborate this, indicating the effect of human intervention in the cropland (Fig. 9). Some possible causes of such a relatively large amount of net carbon uptake in SF are (1) young forest ages (31–50 years), (2) abundant water due to precipitation, (3) relatively weaker disturbance of the summer monsoon during the study period, and (4) warm winter conditions leading to additional photosynthesis in the evergreen forest canopy in winter. Only a few sites in Asia have recorded annual NEE values that are comparable to the cropland and forest sites in this study: (1) − 0.64 kg C m^−2^ year^−1^ in a mixed forest in Japan (34.7833°N, 135.8500°E; no climate information) [70], (2) − 0.83 kg C m^−2^ year^−1^ in a mixed forest in Japan (34.7333°N, 134.3667°E; annual precipitation 1078 mm year^−1^, annual mean T_air_ 15.3°C) [71], and (3) − 0.64 kg C m^−2^ year^−1^ in a fertilized rangeland in Japan (36.9167°N, 139.9667°E; 1561 mm year^−1^, 12.0 °C) [72].

## Conclusions

This study analyzes eddy covariance measurements of CO_2_ fluxes at the land–atmosphere interface across an urbanization gradient in Korea with high-rise high-density urban residential, suburban, double-cropping cropland, and forest canopy areas. Our study shows that the systematic differences due to different data processing for the eddy covariance method are negligible, and the random flux error follows a double exponential distribution even in urban areas. Notably, the relative random flux errors in the urban-influenced areas are larger than those in the forest and cropland, indicating that the direct measurement of CO_2_ fluxes is more challenging in urban areas than in natural vegetation or cropland.

Our analysis demonstrates that the urban residential and suburban areas are constant CO_2_ sources throughout the year, but cropland and SF areas are strong CO_2_ sinks. The carbon uptake by the cropland is comparable to the SF because of the suitable climate and double-crop rotation during the study period. Our study also demonstrates that vegetation at all the sites responds to the summer monsoon and influences seasonal changes in the strengths of carbon sources and sinks. The heavy rain spells during the summer season influence all the sites by decreasing the photosynthetic carbon uptake due to the reduction of solar radiation (i.e., mid-season depression), which has been reported in natural vegetative canopies in this region. Furthermore, the diurnal and seasonal variations of net CO_2_ exchanges are also modulated in urban and suburban areas, just as they are in the cropland and forest canopy along the seasonal progression of the East Asian summer monsoon. Indeed, net CO_2_ flux in the urban and suburban sites increases during the summer monsoon season with the depression of carbon uptake in the monsoon season as urban vegetation responds to the monsoon climate.

The magnitudes of net CO_2_ emission and their temporal dynamics show differences between these two urban-influenced sites despite the similar climate conditions. The net CO_2_ emissions per capita in the urban and suburban areas are 0.7 and 4.9 t CO_2_ year^−1^ person^−1^, respectively. These values are smaller than those from an inventory analysis of typical Korean cities, which indicates that vegetation in the urban and suburban areas offsets the fossil fuel emissions of CO_2_, indicating large uncertainties in urban scale anthropogenic CO_2_ emission. The absolute magnitude of net CO_2_ exchange in the high-rise residential area is smaller than those in other urban sites of similar or smaller population density (< 4700 inh km^−2^) and inventory analyses based on fossil fuel emissions (e.g., [7, 48, 49, 51]). We speculate that this small value is related to the CO_2_ mitigation of urban vegetation and to the district heating system. Despite the high vegetation fraction and scattered buildings, the net CO_2_ emission per capita in the suburban area is considerably larger than in the high-rise high-density residential area in this study and other cities of similar vegetation fraction, possibly because of the heavy traffic volume and factories around the suburban site.

The limitations of this study notwithstanding, our findings have important policy implications for urban regeneration and energy consumption in East Asia, where rapid urbanization has been progressing for the last several decades, by indicating changes in CO_2_ emission across the urbanization gradient and their controlling factors. Importantly, the urban and suburban area has a much larger relative random flux uncertainty than the other sites and previously reported vegetative canopies.

It is notable that urban vegetation mitigates anthropogenic CO_2_ emissions and is influenced by the monsoon activity like natural vegetation in this region. Fossil fuel CO_2_ emission data from inventory have non-negligible differences and currently several megacity carbon projects are focusing on high-resolution mapping of CO_2_ and verification of inventory data (e.g., [61, 73, 74]). Eddy covariance method has been widely used in such urban-focused projects for benchmarking and verification of fossil fuel CO_2_ emissions. Our findings indicate that even in situ flux observation is challenging because of its larger random uncertainty and this larger uncertainty should be carefully considered in urban studies. Also, it is likely that a potential change in urban vegetative carbon uptake in this region might occur in response to the intensification and lengthening of the heavy rain spells in the summer growing season. Further long-term monitoring of CO_2_ fluxes should be conducted with different land cover types in the East Asia region to improve our understanding of the impacts of rapid urbanization and vegetation on the carbon balance.

## Data Availability

All data are available upon request to corresponding author (jhong@yonsei.ac.kr).

## Abbreviations

ANN: artificial neural network
CP: double cropping cropland site
F_C_: CO_2_ flux
HU: high-rise residential urban site
inh: inhabitants
NEE: net ecosystem exchange of CO_2_
P: population density
PAR: photosynthetically active radiation
PDF: probability density function
RH: relative humidity
SF: subtropical forest site
SU: suburban site
T_air_: air temperature
ε: random error
